# Network Pharmacology-Based Exploration of the Mechanism of Action of Shugan Hewei Recipe in the Treatment of Gastroesophageal Reflux Disease with Anxiety and Depression

**DOI:** 10.1155/2022/3957084

**Published:** 2022-09-27

**Authors:** Tingting Xu, Chunfang Liu, Xiulian Zhang, Lin Geng, Hongwei Wang, Li Li, Shengliang Zhu

**Affiliations:** ^1^Yueyang Hospital of Integrated Traditional Chinese and Western Medicine, Shanghai University of Traditional Chinese Medicine, Shanghai 200437, China; ^2^Shanghai Baoshan District Hospital of Integrated Traditional Chinese and Western Medicine, Shanghai 201999, China; ^3^Shanghai Guanghua Hospital of Integrated Chinese and Western Medicine, Shanghai 200050, China; ^4^Institute of Arthritis Research in Integrative Medicine, Shanghai Academy of Traditional Chinese Medicine, Shanghai 200050, China

## Abstract

The Shugan Hewei recipe (SHR) is a well-recognized traditional Chinese medicine (TCM) prescription that has been shown to significantly improve chest pain, acid regurgitation, and the mood of GERD. Nonetheless, the underlying mechanisms remain unclear. In this study, the active compounds and targets of SHR were predicted using network pharmacology. Gene ontology (GO) and Kyoto Encyclopedia of Genes and Genomes (KEGG) analyses were utilized to explore the therapeutic mechanism of SHR. Combined with the drug target obtained from network pharmacology, the therapeutic effect and mechanism of SHR were observed. SHR's main active compounds included quercetin, kaempferol, and luteolin. The core targets of SHR and GERD were TGF-*β*1, IL-1*β*, IL-4, CXCL10, MAPK1, MAPK3, CXCL8, IL-10, IL-2, and FOS, involving virus infection, inflammatory response, and body immunity. The core targets of SHR during the treatment of mental disorders were GABR_A1_, GABR_A2_, GABR_A3_, GABR_A5_, and GABR_A6_, involving synaptic transmission and transmembrane movement. Animal experiments revealed that SHR could repair the lower esophageal mucosa, mediate inflammatory factors, and GABA receptors and improve the behavior of rats. Overall, our results substantiate that SHR has huge prospects for widespread application in treating GERD subjects with anxiety and depression.

## 1. Background

Gastroesophageal reflux disease (GERD) is a common upper gastrointestinal disease with varying incidence worldwide, with high and low incidences in Western countries (18.1–27.8% in North America and 8.8%–25.9% in Europe) and Asia (2.5%–7.8% in East Asia), respectively. However, the increase in global incidence is widely attributed to lifestyle changes [1]. GERD is characterized by frequent attacks, with approximately 13.3% of GERD patients suffering from symptoms such as heartburn and acid reflux every week [2]. Long-term repeated attacks affect people's quality of life, increasing the burden on the healthcare system and medical resources [3].

Proton pump inhibitors (PPIs) are conventionally used to treat GERD to reduce esophageal mucosa damage by inhibiting gastric acid secretion. Nevertheless, disease-associated symptoms are still found in up to 54.1% of GERD patients treated with standard doses of PPIs. Meanwhile, PPIs have obvious side effects, including an increased risk of fracture and altered gut microbiota-induced intestinal infection following long-term use [4, 5]. Little is currently known about the exact mechanism of GERD. In addition to acid-induced esophageal mucosal damage, mental disorders such as anxiety and depression have been documented to exert a crucial role in the pathogenesis of GERD. Current evidence suggests that patients with anxiety and depression still suffer from symptoms after acid suppression, with no significant improvement in their quality of life [6, 7]. Therefore, there is an urgent demand for drugs with satisfying efficacy and low side effects.

Traditional Chinese medicine (TCM) has been used for thousands of years to treat and prevent diseases in China. Shugan Hewei recipe (SHR) is a modified classic famous prescription (Patent NO. ZL201110287443 X) that brings significant benefits in treating GERD accompanied by anxiety, depression, and other mental diseases. SHR is composed of *Bupleurum Falcatum* L. (Chinese name: Chaihu, 9g), *Cyperus Rotundus* L. (Chinese name: Xiangfu, 12g), *Citrus Aurantium* L. (Chinese name: Zhike, 12g), *Corydalis Yanhusuo* (Chinese name: Yanhusuo, 9g), *Inula Japonica Thunb* (Chinese name: Xuanfuhua, 12g), *Haematitum* (Chinese name: Daizheshi, 15g), *Coptis Chinensis Franch* (Chinese name: Huanglian, 3g), *Tetradium Ruticarpum* (Chinese name: Wuzhuyu, 3g), *Zingiber Officinale Rosc* (Chinese name: Shengjiang, 3g), *Polygonatum Sibiricum Red (*Chinese name: Huangjing, 15g), *Gardenia Jasminoides j. Ellis* (Chinese name: Shanzhizi, 9g), *Pteria Martensii (Dunker)* (Chinese name: Zhenzhumu, 15g), and *Concha Arcae* (Chinese name: Duanwalengzi, 30g). An increasing body of evidence suggests that SHR could significantly improve chest pain and acid regurgitation in patients with GERD, relieve anxiety and depression, and reduce the damage to the lower esophagus mucosa of GERD rats [8–10]. Due to the variety of TCM compounds and multipathway, multitarget characteristics of their action mechanisms, the molecular mechanism underlying the efficacy of SHR in treating GERD accompanied by mental disorders remains largely unknown, warranting further exploration.

Importantly, network pharmacology can assist in elucidating the scientific connotation of Chinese medicine prescriptions via visualization of the interactions between the drug component and the target. In light of this concept, network pharmacology was employed in this study for prediction, and animal experiments were conducted to validate our findings and provide novel insight into the specific mechanism of SHR in treating GERD with mental disorders.

## 2. Materials and Methods

### 2.1. Prediction and Screening of Action Targets of SHR

The Traditional Chinese Medicine Systems Pharmacology database and analysis Platform (TCMSP, https://tcmsp-e.com) was adopted to retrieve the targets of the main components of the SHR [9]. According to the absorption, distribution, metabolism, and excretion (ADME) attribute values: oral availability (OB ≥30%) and drug-like activity (DL ≥0.18), the action targets of components were screened to obtain active compounds and protein targets. Additionally, other potential action targets of components were retrieved from the literature. Meanwhile, the PubChem database (https://pubchem.ncbi. nlm.nih.gov) was utilized to generate molecular structure diagrams (SDF format), and the SwissADME database (http://www.swissadme.ch) was utilized to screen compounds based on the criteria: “High” gastrointestinal absorption, and “Yes” for 2 or more of the 5 drug-like activity predictions. The eligible compounds were selected utilizing the Swiss Target Prediction database (http://www.swiss target prediction.ch) to obtain the compound targets [11, 12]. Finally, the UniProt protein database (https://www.uniprot.org) was employed to convert protein targets into corresponding gene targets.

### 2.2. Screening of Therapeutic Targets of GERD with Anxiety and Depression

First, “gastroesophageal reflux disease,” “reflux esophagitis,” “nonerosive reflux disease,” “anxiety,” “depression,” and “mental illness” related to GERD and mental disorders were utilized as keywords to search potential disease targets from the databases including OMIM (https://omim.org), GeneCards (https://www.genecards.org), and Drugbank (https://go.drugbank.com) [13–15]. The screened targets from the aforementioned 3 databases were intersected to obtain the therapeutic targets for GERD and mental disorders after removing the overlapping ones.

### 2.3. Drug-Active Compounds-Target Network of SHR

The “drug-active compounds-target network” was constructed and analyzed by Cytoscape 3.8.0. The SHR, active compounds, and action targets were input to construct the network, where “nodes” represent drugs, active compounds, and action targets, and “edges” represent the relationship between the two. The characteristics involving degree, betweenness degree, closeness degree, etc., were analyzed by Network Analyzer to explore the relationship between active compounds and targets.

### 2.4. Protein-Protein Interaction (PPI) Network of SHR-Disease Targets

The screened targets of SHR compounds were intersected with the targets related to GERD and mental disorders, and a Venn plot was generated. The STRING database (https://www.string-db.org) was used to construct a PPI network of the intersected targets, with the biological category set to “*Homo sapiens”* and the minimum interaction threshold to “highest confidence” (>0.9) [16]. The Cytoscape plug-in MCODE was used to determine the tightly connected areas (modules), screen potential core targets, and describe their function. A module is a complex composed of multiple proteins with close interactions and can more accurately reflect the action mechanism of drug therapy.

### 2.5. Annotation of Pathways

To identify pathways related to the key targets, Gene Ontology (GO) and Kyoto Encyclopedia of Genes and Genomes (KEGG) enrichment analyses on the modules generated by the MCODE tool were carried out using the Metascape database (https://metascape.org) [17].

### 2.6. Animals

Fifty specific pathogen-free male Sprague Dawley (SD) rats weighing (250 ± 50) g were provided by Shanghai SLAC Lab Animal Company Ltd. (Shanghai, China). The rats were housed in the animal center of Yueyang Hospital of Integrated Traditional Chinese and Western Medicine, Shanghai University of Traditional Chinese Medicine (Animal License Number: SYXK (Hu) 2018-0040) at a temperature of (20 ± 5)°C, with humidity of (50 ± 10)%, 12 h/12 h light/dark cycle, with ad libitum access to food and water. Rats were used for modeling after one week of acclimatization.

### 2.7. Animal Modeling and Drug Treatments

The SD rats were randomly assigned to the sham operation group (SO group), composite model group (acid reflux model + psychological stress model, CM group), Shugan Hewei recipe group (SHR group), and omeprazole group (OM group). Rats in the CM group were subjected to pylorus semiligation combined with cardia muscle tearing to establish a GERD model as previously described by Tugay et al. [18]. Given that infection is easily induced and mortality may be increased by psychological stress immediately after modeling, psychological stress was induced 7 days after surgery. The chronic unpredictable mild stress (CUMS) model is widely acknowledged as the most effective model for induction of psychological stress [19, 20] and mainly includes forced swimming test, restraint test, day and night reversal, fasting, and water deprivation. The induction of psychological stress was conducted for 14 consecutive days, with one of the aforementioned stimuli randomly selected every day. The rats in the SO group only underwent laparotomy. 7 days after the operation, rats were given corresponding drugs before the daily psychological stress induction.

SHR was purchased from Shanghai Leiyunshang Traditional Chinese Medicine Decoction Piece Factory Co., Ltd. The batch numbers of SHR are as follows: 1810079 *(Bupleurum Falcatum* L), 1806052 *(Cyperus Rotundus* L), 1805167 (*Citrus Aurantium* L), 1807080 *(Corydalis Yanhusuo)*, 1809036 *(Inula Japonica Thunb)*, 1805087 *(Haematitum)*, 180922 *(Coptis Chinensis Franch)*, 1807114 *(Tetradium Ruticarpum)*, 1806047 *(Zingiber Officinale Rosc)*, 1806090 (*Polygonatum Sibiricum Red)*, 1801042 (*Gardenia Jasminoides j. Ellis)*, 1806073 (*Pteria Martensii (Dunker),* and 1804097 *(Concha Arcae*). The rats in the SO and CM groups were given 1.5 mL of normal saline every day, those in the SHR group 1.5 mL of TCM every day, and those in the OM group 1.5 mL of omeprazole aqueous solution every day. The peripheral blood and tissue samples were collected after 14 days of psychological stress induction.

### 2.8. Behavioral Observation

#### 2.8.1. Sucrose Preference Test (SPT)

The SPT was performed to observe the changes in anxiety and depression in rats before psychological stress induction and after 14 days. Specifically, within the first 24 h, two bottles loaded with 1% sucrose water of equal volume were placed in each cage at the same time, and within the second 24 h, the sucrose water was replaced with pure water in the one bottle to allow the rats to be more familiar with sucrose water. Over the third 24-h period, the rats were deprived of water. Equal volumes of 1% sucrose water and pure water were given for the next 24 h. Finally, the consumption of sucrose water and pure water was measured 1 h later, and the percentage of sucrose water intake was calculated [21].

#### 2.8.2. Open Field Test (OFT)

An OFT was conducted before psychological stress induction and after 14 days. The activity of the rats in the open field (40 cm in height × 80 cm in diameter) over a 5-min period was recorded by a video camera. Fewer grids crossed, shorter standing times, and longer grooming times were indicative of a more tense status of the rats. After the experiment, a video-tracking system (VideoMot2, German TSE Company, German) was utilized for data analysis [22, 23].

#### 2.8.3. Forced Swimming Test (FST)

The forced swimming test was performed before psychological stress induction and after 14 days. The immobility time (s) of the rats within 5 min was observed to assess the helplessness of the rats. After a period of stress induction, despair/hopelessness was observed, leading to prolonged immobility time. Indeed, the forced swimming test is commonly used to assess depression in rats [24].

### 2.9. Peripheral Blood and Tissue Collection

Samples were collected 21 days after modeling. After pentobarbital anesthesia, 4 mL of blood was harvested from the abdominal aorta and centrifuged at 3500 r/min for 10 min. Subsequently, the supernatant was extracted and stored in an Eppendorf (EP) tube at −80°C for later use. The esophageal tissues (1 cm in length) at the upper margin of the cardia were split longitudinally and then sliced longitudinally into 2 portions along the midline. One portion was fixed in neutral formaldehyde, and the other was placed in an EP tube and frozen at −80°C for later use. The whole brain was collected, and the hypothalamus was isolated using curved forceps and preserved in an EP tube at −80°C for later use.

### 2.10. Hematoxylin and Eosin (HE) Staining

The lower esophageal tissues were fixed in neutral formaldehyde for 24 h, dehydrated, and embedded in paraffin. The tissues were sliced into sections that were stained with hematoxylin and eosin. The pathological changes in the lower esophagus mucosa were observed under a light microscope.

### 2.11. Enzyme-Linked Immunoassay (ELISA)

According to the ELISA kit, the contents of IL-4 and IL-10 proteins (96-well, BOSTER Biological Technology, Wuhan, China) and IL-8 protein (96-well, Shanghai Yuanye Bio-Technology Co., Ltd., China) in the peripheral blood of rats were measured. The absorbance was recorded on a microplate reader (BioTeK Instruments, Winooski, VT, USA) at the wavelength of 450 nm.

### 2.12. RT-PCR Determination

RNA was extracted from frozen tissues, and the RNA concentration was measured. After reverse transcription, the cDNA was generated. The final reaction system (10 *μ*L) consisted of 5 *μ*L SYBR GREEN (2 × , Rocher), 0.2 *μ*L forward primer, 0.2 *μ*L reverse primer, 2.6 *μ*L H_2_O, and 2 *μ*L cDNA. Three parallel wells were set for each sample to avoid sampling error. The primers used were as follows.

#### 2.12.1. Ethics Statement

This study was approved by the animal ethics committee of Yueyang Hospital of Integrated Chinese and Western Medicine, affiliated with the Shanghai University of Traditional Chinese Medicine (Animal Use Summary 16634). It is well established that intraperitoneal injection of pentobarbital sodium can reduce injury in rats.

### 2.13. Statistical Analysis

Data were analyzed using SPSS 24.0 statistical software and expressed as mean ± standard deviation ($\bar{x}$ ± *s*). Comparison between two groups was conducted using a *t*-test and among multiple groups achieved by one-way analysis of variance (ANOVA). A *p* value <0.05 was statistically significant.

## 3. Results

### 3.1. Active Compounds and Targets of SHR

**Figure 1:**
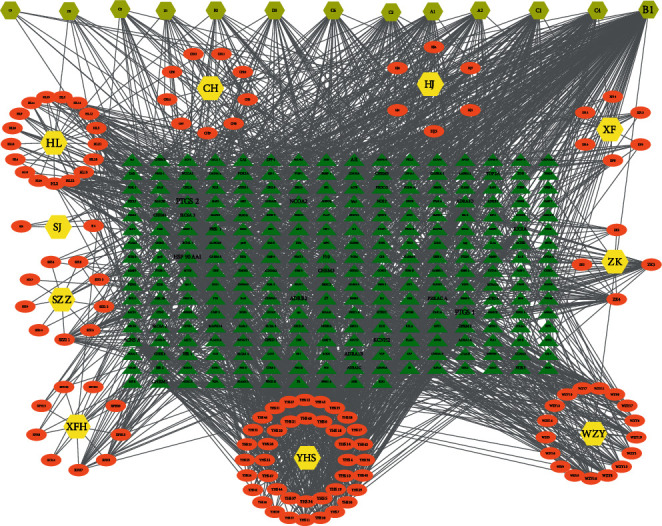
The drugs-active compounds-target interaction network of SHR. *Note.* In this network, the circle indicates the active compounds of the drug, the rectangle indicates the target, the diamond indicates the drug name, and the font size represents the degree value; ZK (zhi ke); XF (Xiang Fu); CH (Chai Hu); SJ (Sheng Jiang); XFH (Xuan Fu Hua); HJ (Huang Jing); HL (Huang Lian); WZY (Wu Zhu Yu); SZZ (Shan Zhizi); YHS (Yan Husuo); A1 refers to the intersection among SJ, HJ, XFH, SZZ, and ZK; A2 refers to the intersection among SJ, XF, CH, YHS, and SZZ; B1 refers to the intersection among WZY, XF, CH, HL, XFH, and SZZ; B2 refers to the intersection among WZY, HL, and YHS; C1 refers to the intersection between RXF and XFH; C2 refers to the intersection among XF, CH, and XFH; C3 refers to the intersection among HJ and YHS; C4 refers to the intersection among XF, CH, YHS, and SZZ; C5 refers to the intersection between XF and XFH; C6 refers to the intersection between XFH, XF, and YHS; D1 refers to the intersection between HL and YHS; D2 refers to the intersection between HL and YHS.

A total of 196 active compounds were identified in SHR. After intersecting the screened results, 144 active compounds and 3398 targets were obtained. Next, the overlapping targets were deleted after merging, and 266 targets were obtained. Cytoscape 3.8.0 was utilized to generate a drugs-active compounds-target interaction network of SHR, which contained 410 nodes and 2244 edges. NetworkAnalyzer in Cytoscape suggested that quercetin had a degree value of 166, a betweenness centrality of 0.406682063, and a closeness centrality of 0.536939314. Therefore, quercetin was predicted to be the main compound of SHR in treating GERD with anxiety and depression. In addition to quercetin, kaempferol (degree value of 63, betweenness centrality of 0.056429186, and closeness centrality of 0.42219917) and luteolin (degree value of 59, betweenness centrality of 0.0665468, and closeness centrality of 0.416155419) were also the main compounds of SHR. Quercetin is mainly distributed in *Bupleurum*, *Rhizoma Cyperi*, *Inula Flower*, *Coptis, Evodia,* and stir-baked Gardenia, while kaempferol is mainly distributed in *Bupleurum, Rhizoma Cyperi, Inula Flower*, and stir-baked Gardenia (Table 1, Figure 1).

### 3.2. Targets Related to GERD with Mental Disorders

Up to now, research on GERD with anxiety and depression has mainly focused on clinical observation, with few studies emphasizing research of the underlying mechanism. To explore the therapeutic effect of SHR more comprehensively, using network pharmacology, GERD and mental diseases were divided into two groups to explore the effect of SHR on these two diseases. Based on the predicted results from GeneCards, OMIM, and Drug bank databases, 1959 targets related to GERD (containing RE and NERD) and 1367 targets related to mental disorders (including anxiety and depression) were identified.

### 3.3. PPI Network of SHR Active Compounds-Disease Targets

#### 3.3.1. PPI Network of SHR Active Compounds-GERD Targets

**Figure 2:**
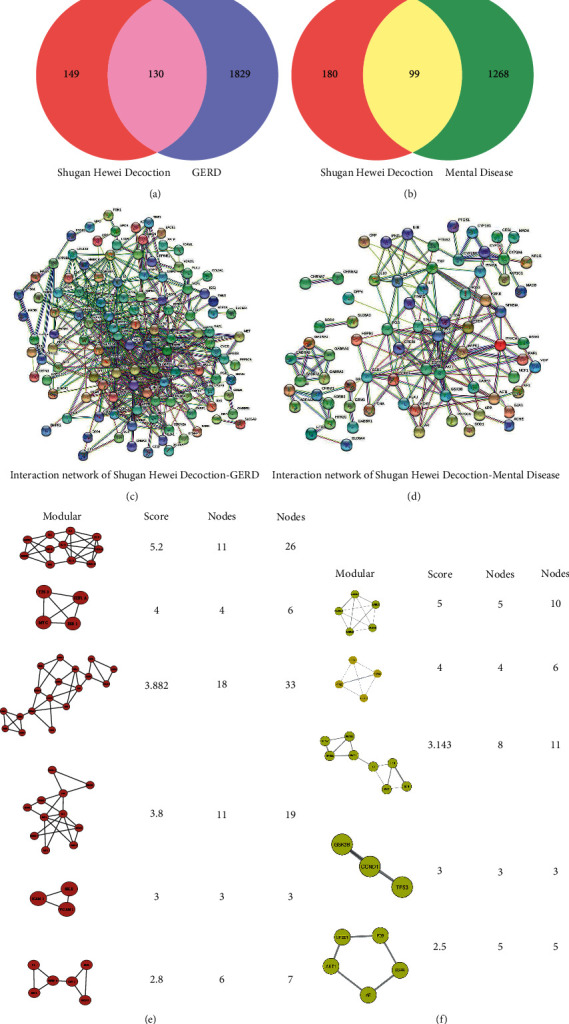
The PPI network of SHR active compounds-disease targets. Note: (a) Venn diagram of SHR compounds targets and GERD-associated targets. (b) Venn diagram of SHR compounds targets and targets related to mental disorders. (c) PPI network of SHR active compounds-GERD targets. (e) The module network of SHR active compounds-GERD core targets. (d) PPI network of SHR-mental disorder intersection targets. (f) The module network of SHR active compounds-mental disorders core targets.

The SHR compound targets and GERD-associated targets were intersected, yielding 130 common targets in the Venn plot (Figure 2(a)). Next, the PPI network of SHR-GERD common targets was constructed using the STRING database and subsequently analyzed with the MCODE plug-in in Cytoscape 3.8.0 software. The generated modules were sorted according to their score values, and the potential core targets TGF-*β*1, IL-1*β*, IL-4, CXCL10, MAPK1, MAPK3, CXCL8, IL-10, IL-2, and FOS were obtained (Figure 2(c), 2(e)).

#### 3.3.2. PPI Network of SHR Active Compounds-Mental Disorders Targets

The SHR compounds targets and targets related to mental disorders were intersected, yielding 99 common targets in the Venn plot (Figure 2(b)). STRING database was used to construct the PPI network of SHR-mental disorder common targets. This network was further analyzed using the MCODE plug-in in Cytoscape 3.8.0 software to identify modules sorted according to their score values. Finally, the putative core targets GABR_A1_, GABR_A2_, GABR_A3_, GABR_A5_, and GABR_A6_ were determined (Figures 2(d) and 2(f)).

### 3.4. In Silico Analysis of Targets

GO biological process and KEGG pathway enrichment analyses on the modules classified by MCODE in the PPI network of SHR-GERD and SHR-mental disorder targets were conducted with Metascape software. Biological process (BP), molecular function (MF), and cell component (CC) were included in the GO annotations.

#### 3.4.1. GO and KEGG Analyses on SHR-GERD Targets

**Figure 3:**
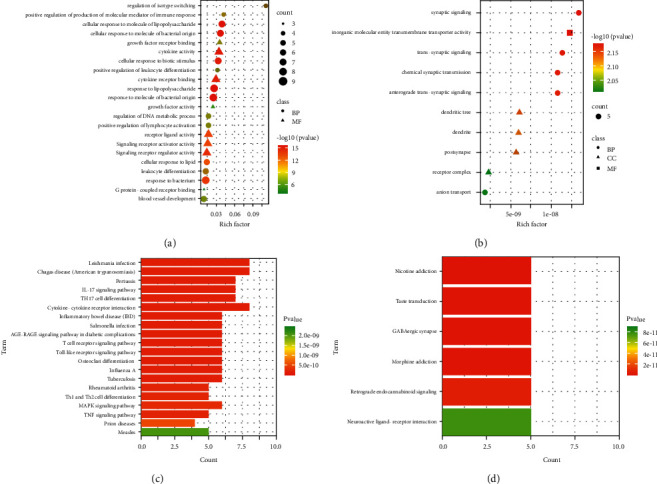
GO and KEGG analyses on the SHR-GERD and SHR-mental disorder targets. Note: (a) Bubble charts of SHR action targets in the treatment of GERD. (b) Bubble charts of SHR action targets in the treatment of mental disorders. (c) Pathways enriched by SHR action targets in the treatment of GERD. (d) Pathways enriched by SHR action targets in the treatment of mental disorders.

The GO analysis of SHR-GERD targets showed 345 entries in BP (333 of which with *p*-value <0.01) and 8 entries in MF with *p*values <0.01 (Table 2). The top 20 entries in BP and 8 entries in MF were visualized in bubble plots. We found that SHR-related targets were significantly enriched in BP, including response to lipopolysaccharide, response to molecule of bacterial origin, cellular response to lipopolysaccharide, cellular response to molecule of bacterial origin, and cellular response to biotic stimulus. The action targets of SHR for treating GERD correlated with MF, including cytokine activity, cytokine receptor binding, receptor-ligand activity, signaling receptor activator activity, and signaling receptor regulator activity (Figure 3(a)).

KEGG analysis of the SHR-GERD targets gained from the Metascape database yielded 62 pathways. The top 20 pathways were selected according to the *p*-value. As depicted in Figure 3(c), the SHR action targets in treating GERD were mainly related to the pathways enriched by Leishmania infection, Chagas disease (American trypanosomiasis), pertussis, IL-17 signaling pathway, and Th17 cell differentiation.

#### 3.4.2. GO and KEGG Analyses on the SHR-Mental Disorder Targets

GO analysis revealed 14 SHR-mental disorder entries in BP (5 of which with *p*-value <0.01), 27 entries in CC (4 of which with *p*-value <0.01), and 25 entries in MF (1 of which with *p*-value <0.01) (Table 3). We then generated bubble plots of entries with a *p*-value <0.01, showing that SHR action targets might involve synaptic signaling, trans-synaptic signaling, chemical synaptic transmission, anterograde trans-synaptic signaling, anion transport-associated BP. As for CC, SHR action targets were related to the dendritic tree, dendrite, postsynapse, and receptor complex. Finally, SHR action targets were related to MF, including inorganic molecular entity transmembrane transporter activity (Figure 3(b)).

KEGG analysis was conducted on SHR-mental disorder targets obtained from the Metascape database, yielding 6 pathways (*p*-value <0.01). The data in Figure 3(d) exhibited that the SHR action targets in treating mental disorders were significantly enriched in pathways, including nicotine addiction, taste transduction, GABAergic synapse, morphine addiction, retrograde endocannabinoid signaling, and neuroactive ligand-receptor interaction.

### 3.5. SHR Repairs Lower Esophageal Mucosa Damage in CM Rats

**Figure 4:**
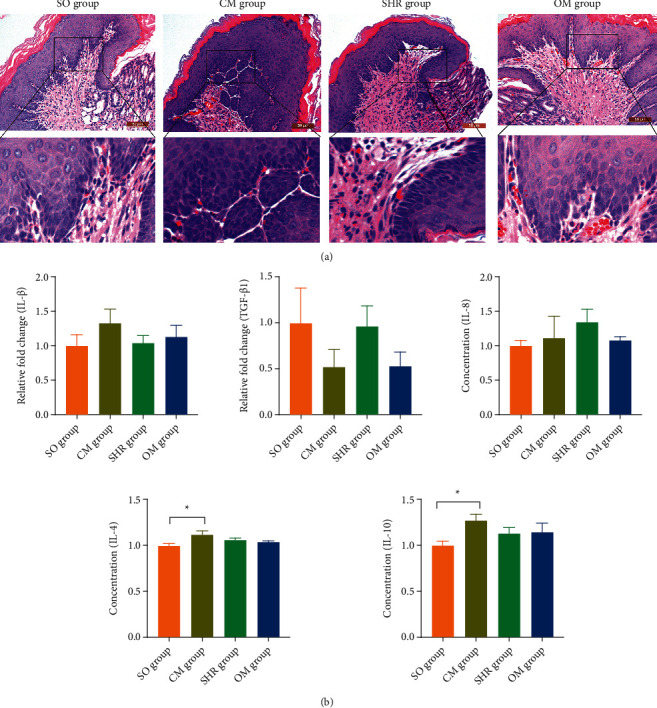
(a) Histopathological changes of the lower esophagus (HE, × 200). (b) Expression of esophageal IL-1*β* and TGF-*β*1 mRNA as well as peripheral serum IL-4, IL-8, and IL-10 proteins in rats of each group. ^*∗*^*p* < 0.05; ^*∗∗*^*p* < 0.001.

HE staining exhibited a significantly curved basal layer, increased and multiple layers of prickle cells, increased papilla, and numerous vascular lakes in the esophageal mucosa of rats in the CM group. Both SHR and omeprazole could reduce the number of prickle cells in the esophageal mucosa and repair the basal layer and stratum corneum (Figure 4(a)).

### 3.6. SHR Potentially Mediates Inflammation in CM Rats

We found that the mRNA levels of IL-1*β* and TGF-*β*1 mRNA and the protein expression of IL-8 and IL-10 were not elevated. However, IL-4 levels were raised in the CM group (*p* < 0.05). After treatment with either SHR or omeprazole, the contents of IL-4 and IL-10 were mildly reduced (*p* > 0.05) (Figure 4(b)).

### 3.7. SHR Upregulates the Expression of GABA Receptors in the Hypothalamus in CM Rats

**Figure 5:**
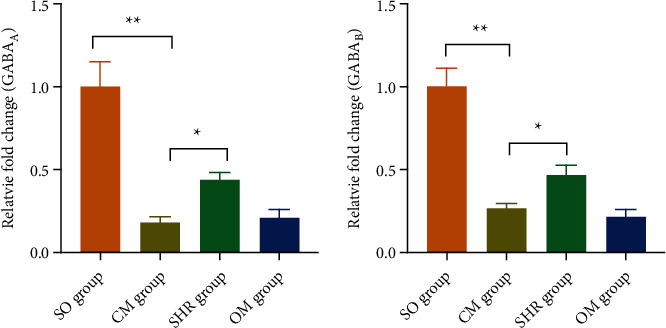
Expression GABA receptors in hypothalamus, including GABA_A_ and GABA_B_, ^*∗*^*p* < 0.05, ^*∗∗*^*p* < 0.001.

The expression of inhibitory neurotransmitters GABA_A_ mRNA and GABA_B_ mRNA was decreased in the hypothalamus of the CM rats (*p* < 0.001). Treatment with SHR upregulated the levels of GABA_A_ mRNA and GABA_B_ mRNA (*p* < 0.05) and restored the anxiety and depression of model rats to a normal status. In contrast, omeprazole could not upregulate the levels of GABA_A_ mRNA and GABA_B_ mRNA (Figure 5).

### 3.8. SHR Improves the Behavior of CM Rats

**Figure 6:**
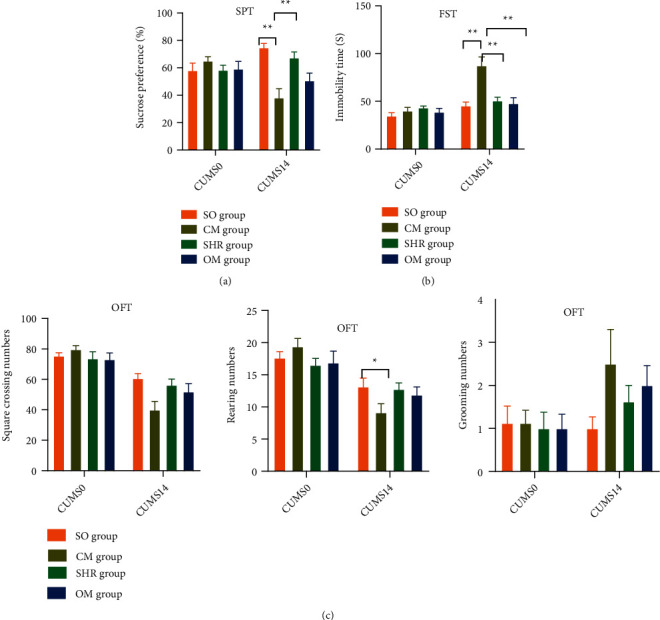
Behavioral testing. Behavioral changes of rats in different groups in CUMS0 and CUMS14, including sucrose preference test (a), forced swimming test (b), and open field test (c). ^*∗*^*p* < 0.05; ^*∗∗*^*p* < 0.001.

The results of behavioral tests displayed reduced sucrose preference in CM rats. During the OFT, the number of grids crossed and rearing times were reduced, but the grooming times increased, exhibiting reduced exploration of new environments. Additionally, the immobility time of the CM rats was prolonged in the FST, indicative of a despair-like state. SHR treatment contributed to enhanced sucrose preference of rats (*p* < 0.001), shorter immobility time in the FST (*p* < 0.001), and increased exploration of new environments, which was evidenced by increased grid crossings and reduced grooming times (*p* > 0.05). Omeprazole could also shorten the immobility time in FST (*p* < 0.001) but not change the sucrose preference and exploration of new environments (Figure 6).

## 4. Discussion

According to TCM theory, GERD is related to the liver and stomach. Mental disorders, liver qi depression, failure of the liver's free coursing, qi counterflow, and impaired normal descending of stomach qi, among others, result in heartburn and acid reflux. This theory is generally similar to western medicine, emphasizing mental illness's impact on GERD and the patient's perception of symptoms [25]. TCM prescriptions, including Chaihu Shugan powder, Xuanfu Daizhe decoction, and Zuojin pill, have demonstrated a striking clinical efficacy in the treatment of mental disorders and gastrointestinal diseases with minimal side effects, which highlights their potential for the treatment of GERD accompanied by anxiety- and depression-like mental disorders [26, 27]. An increasing body of evidence suggests that bupleurum liver-coursing powder originated from Jing Yue Quan Shu (Jing-Yue's Complete Compendium), can dredge the liver, rectify the qi, quicken the blood, and relieve the pain, demonstrating good clinical efficacy for anxiety- and depression-like mental disorders and digestive system diseases [28, 29]. Xuanfu Daizhe decoction, derived from differentiating and treating the Tai Yang disease syndrome, treatise on cold damage, is commonly used to treat GERD. The Zuojin pill, described in the Chinese text Dan Xi Xin Fa, has been substantiated to have good clinical efficacy for treating gastrointestinal inflammation [30]. SHR's efficacy is primarily attributed to quercetin, kaempferol, and luteolin, and its significant long-term clinical efficacy has been demonstrated in clinical practice. Nevertheless, the multiple-target action mechanisms of SHR remain largely obscure, emphasizing the need for further studies. Therefore, network pharmacology was adopted in this study to probe the potential targets of the SHR in treating GERD with mental illness, and animal experiments were carried out to verify its effectiveness and the underlying mechanism of action.

Based on the Cytoscape network analysis results, quercetin was predicted to be the major active compound of SHR in treating GERD with anxiety and depression, followed by kaempferol and luteolin. Quercetin, a flavonoid and a neuroprotective agent, can reportedly ameliorate learning and memory impairments, anxiety, and depression triggered by CUMS, improve animal behavior, and protect against nerve damage evoked by neurological diseases or other diseases [31, 32]. Meanwhile, studies have addressed its anti-inflammatory effect. For instance, quercetin has been reported to downregulate the expression of IL-1*β*, IL-6, TNF-*α*, NF-*κ*B, and other pro-inflammatory cytokines, alleviating acute pancreatitis [33]. Kaempferol is also a flavonoid that exerts a powerful anti-inflammatory activity and a protective effect against anxiety and depression symptoms [34, 35]. This finding provides a theoretical basis for SHR in treating GERD accompanied by anxiety and depression, and other mental disorders. We also identified quercetin and kaempferol in Xuanfu Daizhe decoction, bupleurum liver-coursing powder, and Zuojin pill, suggesting that these three have a synergistic effect in the treatment of diseases.

Based on the PPI network and MCODE results, the therapeutic effect of SHR on GERD was mainly mediated by inflammatory cytokines. It has long been thought that esophageal mucosal damage in GERD is caused by acid reflux or protease-induced stimulation in the esophagus. However, the latest research suggests that the esophageal mucosal damage is evoked by chronic chemokines, which is attributed to the infiltration of lymphocytes from the submucosa to the mucosal surface, suggesting the key roles that inflammatory factors play in the pathogenesis of GERD [36, 37]. In the present study, GO and KEGG analyses highlighted that the therapeutic effect of SHR in GERD was inflammation-related. It is well established that Th17 can be produced after the proliferation and differentiation of CD4^+^ T cells stimulated by different antigens. In addition, CD4^+^ T cells differentiate into Th1, Th2, and Treg [38]. It is widely acknowledged that TGF-*β*1 and IL-1*β* drive the differentiation of initial CD4+ T cells and further stimulate the formation of Th17 cells mediated by TNF-*ɑ*, IL-6, and IL-2. IL-17 is a cytokine released from Th17 cells, and Th17 activation induces IL-23 activation, which leads to the secretion of Th17. IL-17/IL-23 affects the differentiation of Th17 by positive feedback [39, 40]. Although IL-17 shows a weak ability to induce inflammation, it can regulate chemokines, such as CXCL1, CXCL2, and IL-8, to maintain the level of inflammation. However, IL-4 and IL-10 are anti-inflammatory factors that can inhibit IL-17 expression, thereby maintaining homeostasis [41, 42]. Combined with the core targets enriched in Th17 and IL-17 pathways, we selected TGF-*β*1, IL-1*β*, IL-8, IL-4, IL-10, and other inflammatory factors to monitor the effect of SHR on GERD. Importantly, we found that although the pro-inflammatory cytokines were not affected, the levels of anti-inflammatory cytokines increased in GERD rats with mental illness. SHR and omeprazole could reduce the protein expression of IL-4 and IL-10, but the difference was not statistically significant. It has been established that long-term psychological stress can cause a Th1/Th2 balance imbalance. Th1 mainly induces an immune response and facilitates the release of inflammatory factors. Th2 mainly mediates humoral immunity, and its abnormal expression is related to hypersensitivity. In the context of psychological stress, the increase in IL-4 boosts Th2 cell differentiation, while the decrease of IL-12 and the increase of IL-10 reduce the number of Th1 cells, thereby reducing the Th1/Th2 ratio and consequently contributing to the transformation of the humoral immune system [43, 44]. This finding may be the main reason for increased anti-inflammatory factors in CM rats.

Herein, we found that GABA-related receptors mainly mediate the effect of SHR in treating mental disorders. GO and KEGG analyses further suggested that the intervention of SHR in mental disorders was principally associated with synaptic transmission, transmembrane movement, and *N*-related pathways. GABA is a crucial inhibitory neurotransmitter in the central nervous system related to depression, epilepsy, drug addiction, and other diseases [45]. It is widely acknowledged that GABA mainly exerts an inhibitory effect through GABA_A_ and GABA_B_ receptors. GABA_A_ and GABA_B_ synergistically exert their inhibitory effect; GABA_A_ affects postsynaptic membrane hyperplasia mediated by ligand-gated chloride channels, while GABA_B_ strengthens the inhibitory signal through *G* protein and second messengers [46, 47]. According to the expression patterns of core targets, GO entries and KEGG pathways, GABA_A_ receptor and GABA_B_ receptor were selected for validation in this study. Our findings suggest that SHR might improve anxiety and depression by mediating neurotransmitter GABA expression. Although omeprazole can inhibit gastric acid secretion and relieve the symptoms caused by acid reflux, it has limited efficacy in relieving anxiety, depression, and other mental disorders.

Meanwhile, this study explored how SHR improved the behaviors of rats with GERD accompanied by anxiety and depression and underscored that SHR significantly increased the sucrose preference of rats, reduced the immobility time during the FST, and increased their exploration of a new environment. Our findings suggest that SHR may regulate GABA receptor or inflammatory factors to relieve anxiety and depression, alleviate symptoms, and lead to normal behaviors in CM group rats. Over the years, the anti-inflammatory and ameliorative effects of TCM have been gradually confirmed, and inflammatory factors have been shown to regulate neurotransmitters [48–52]. Nonetheless, the relationship between inflammatory factors and neurotransmitters in this disease remains unclear, warranting further study.

## 5. Conclusion

The effective active compounds of SHR and the targets for treating GERD with mental diseases such as anxiety and depression were determined through network pharmacology, and the associated pathways were identified through GO and KEGG analyses. The results showed that the main active compounds of SHR were quercetin, kaempferol, luteolin, etc., which mainly acted on the inflammatory pathway and synaptic signal transduction pathway. Based on the network pharmacologic data, our study further verified the therapeutic effects of SHR on GERD with anxiety, depression, and other mental disorders through animal experiments. Accordingly, our results provided compelling evidence that SHR can regulate the immune response, upregulate the inhibitory neurotransmitters in the central nervous system, and restore normal rat behavior.

## Figures and Tables

**Table 1:** The characteristic parameters of SHR active compounds network nodes.

MolID	MolName	Name	Degree	Betweenness centrality	Closeness centrality
MOL000098	Quercetin	B1	166	0.406682063	0.536939314
MOL000422	Kaempferol	C4	63	0.056429186	0.42219917
MOL000006	Luteolin	C1	59	0.0665468	0.416155419
MOL000449	Stigmasterol	A2	45	0.026355551	0.405378486
MOL000358	Beta-sitosterol	A1	42	0.035593182	0.402173913
MOL000354	Isorhamnetin	C2	42	0.021794478	0.402173913
MOL004071	Hyndarin	C6	39	0.016708202	0.399803536
MOL004328	Naringenin	ZK3	34	0.092949086	0.384688091
MOL004215	Leonticine	YHS34	34	0.014886469	0.389846743
MOL005828	Nobiletin	ZK4	34	0.031710737	0.394379845
MOL000790	Isocorypalmine	YHS5	33	0.008486564	0.389846743
MOL002714	Baicalein	HJ5	33	0.048648725	0.393617021
MOL002903	(R)-canadine	D3	32	0.00689397	0.389846743
MOL000141	Hydroxytyrosol	HL2	30	0.026792663	0.386882129
MOL004193	Clarkeanidine	YHS19	29	0.004669128	0.386882129
MOL001460	Cryptopin	YHS10	28	0.007758412	0.386882129
MOL000787	Fumarine	YHS4	28	0.011274499	0.386148008
MOL002670	Cavidine	YHS14	27	0.004206521	0.385416667
MOL004191	Capaurine	YHS18	27	0.004225311	0.385416667
MOL000791	Bicuculline	YHS6	26	0.047536121	0.383962264

**Table 2:** Pathway enrichment analysis of SHR-GERD targets.

GO	Description	Log*p*	Count	Hits
Hsa 05140	Leishmania infection	−18.65	8	FOS, IL-1*α*, IL-1*β*, IL-4, IL-10, MAPK1, MAPK3, TGF-*β*1, CXCL8
Hsa 05142	Chagas disease (American trypanosomiasis)	−17.44	8	FOS, IL-1*β*, IL-2, CXCL8, IL-10, MAPK1, MAPK3, TGF-*β*1
Hsa 05133	Pertussis	−15.59	7	FOS, IL1*α*, IL-1*β*, CXCL8, IL-10, MAPK1, MAPK3
Hsa 04657	IL-17 signaling pathway	−14.95	7	FOS, IL-1*β*, IL-4, CXCL8, CXCL10, MAPK1, MAPK3, IL-2, TGF-*β*1, IL-10, IL-1*α*
Hsa 04659	Th17 cell differentiation	−14.37	7	FOS, IL1*β*, IL-2, IL-4, MAPK1, MAPK3, TGF-*β*1

**Table 3:** Pathway enrichment analysis of SHR action targets in the treatment of mental disorders.

GOact	Description	Log*p*	Count	Hits
Hsa 05033	Nicotine addiction	−14.24	5	GABR_A1_, GABR_A2_, GABR_A3_, GABR_A5_, GABR_A6_
Hsa 04742	Taste transduction	−10.48	5	GABR_A1_, GABR_A2_, GABR_A3_, GABR_A5_, GABR_A6_
Hsa 04727	GABAergic synapse	−12.50	5	GABR_A1_, GABR_A2_, GABR_A3_, GABR_A5_, GABR_A6_
Hsa 05032	Morphine addiction	−12.48	5	GABR_A1_, GABR_A2_, GABR_A3_, GABR_A5_, GABR_A6_
Hsa 04723	Retrograde endocannabinoid signaling	−11.16	5	GABR_A1_, GABR_A2_, GABR_A3_, GABR_A5_, GABR_A6_
Hsa 04080	Neuroactive ligand -receptor interaction	−10.05	5	GABR_A1_, GABR_A2_, GABR_A3_, GABR_A5_, GABR_A6_

## Data Availability

Data that support the findings of this study are available on request.

## Abbreviation

SHR: Shugan Hewei recipe
TCM: Traditional Chinese Medicine
GO: Gene Ontology
KEGG: Kyoto Encyclopedia of Genes
GERD: Gastroesophageal reflux disease
TCMSP: Traditional Chinese Medicines Systems Pharmacology Platform
OB: Oral availability
DL: Drug-like
PPI: Protein-protein interaction
SO group: Sham operation group
CM group: Composite model group
SHR group: Shugan Hewei recipe group
OM group: Omeprazole group
CUMS: Chronic unpredictable mild stress
SPT: Sucrose preference test
OFT: Open field test
FST: Forced swimming test
HEL: Hematoxylin and eosin
ELISA: Enzyme-linked immunoassay
ZK: Zhi Ke
XF: Xiang Fu
CH: Chai Hu
SJ: Sheng Jiang
XFH: Xuan Fu Hua
HJ: Huang Jing
HL: Huang Lian
WZY: Wu Zhu Yu
SZZ: Shan Zhizi
YHS: Yan Husuo
BP: Biological process
MF: Molecular function
CC: Cell component
GABA: Gamma-aminobutyric acid.
